# Dynamic mesolimbic dopamine signaling during action sequence learning and expectation violation

**DOI:** 10.1038/srep20231

**Published:** 2016-02-12

**Authors:** Anne L. Collins, Venuz Y. Greenfield, Jeffrey K. Bye, Kay E. Linker, Alice S. Wang, Kate M. Wassum

**Affiliations:** 1 Dept. of Psychology, UCLA, Los Angeles, CA 90095, USA; 2 Brain Research Institute, UCLA, Los Angeles, CA 90095, USA

## Abstract

Prolonged mesolimbic dopamine concentration changes have been detected during spatial navigation, but little is known about the conditions that engender this signaling profile or how it develops with learning. To address this, we monitored dopamine concentration changes in the nucleus accumbens core of rats throughout acquisition and performance of an instrumental action sequence task. Prolonged dopamine concentration changes were detected that ramped up as rats executed each action sequence and declined after earned reward collection. With learning, dopamine concentration began to rise increasingly earlier in the execution of the sequence and ultimately backpropagated away from stereotyped sequence actions, becoming only transiently elevated by the most distal and unexpected reward predictor. Action sequence-related dopamine signaling was reactivated in well-trained rats if they became disengaged in the task and in response to an unexpected change in the value, but not identity of the earned reward. Throughout training and test, dopamine signaling correlated with sequence performance. These results suggest that action sequences can engender a prolonged mode of dopamine signaling in the nucleus accumbens core and that such signaling relates to elements of the motivation underlying sequence execution and is dynamic with learning, overtraining and violations in reward expectation.

Considerable evidence suggests that midbrain dopamine cell activity^1^,^2^,^3^,^4^ and phasic striatal dopamine release^5^,^6^ closely correlate with the reward-prediction error term proposed by temporal difference reinforcement learning theories to mediate some forms of associative learning. These studies have primarily (though not exclusively^7^,^8^,^9^,^10^) focused on scenarios in which reward follows close in time and space from the stimuli or actions that precede it. A recent study investigated dopamine signaling during procurement of remote reward and found that dopamine concentration in the ventromedial striatum gradually increased as rats navigated a maze, peaking at the time the reward goal was reached^11^. Tonic dopamine changes have been investigated with microdialysis for over 20 years, with results indicating a slow rise in mesolimbic dopamine levels during reward seeking^12^,^13^,^14^,^15^. The recently reported prolonged dopamine concentration increases^11^ were, however, too short lived (~10 s) to be considered tonic, but were also more sustained than typical phasic dopamine signals. To help understand this prolonged form of dopamine signaling we sought to establish additional conditions that engender such signaling and to determine whether prolonged dopamine elevations are dynamic with learning and expectation violation.

It was suggested that prolonged dopamine concentration changes could provide the sustained motivational drive required to navigate through space to distal rewards, because, among other findings, dopamine concentration scaled flexibly with distance to reward^11^. Indeed, ventral striatal dopamine signaling has long been implicated in motivation^16^,^17^,^18^. Sustained motivation is not exclusive to spatial navigation. Instrumental action sequences provide another prevalent situation wherein persistent motivation is required to drive behavior from initial actions through a series of events to earn a distal reward. Therefore, here we evaluated whether dopamine in the nucleus accumbens core (NAc) would show a prolonged signaling profile during the execution of an instrumental action sequence to earn food reward.

On the basis of our previous between-subject evaluation of mesolimbic dopamine signaling during execution of an instrumental sequence^7^, we anticipated fluctuations in the profile of action sequence-related dopamine signaling with learning. Here we used a within-subject, longitudinal recording approach and found evidence of prolonged dopamine concentration elevations during action sequence performance and fluctuations in this signaling with learning. With extended training, dopamine signaling was found to backpropagate away from action execution to the most distal and unexpected predictor of reward, but was reengaged by an unexpectedly higher value, but not different identity, reward.

## Results

### Action sequence task and performance

**Figure 1:**
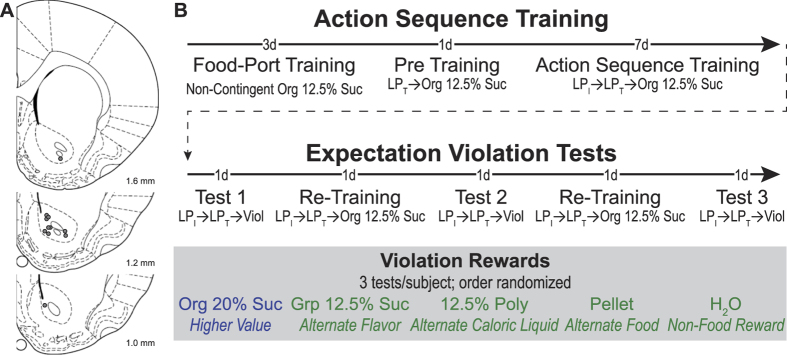
Histological verification of recording sites and experimental design. (**A**) Coronal section drawings taken from^79^. These figures were published in *The rat brain in stereotaxic coordinates*, 4^th^ edn, Paxinos & Watson, 175–177, Copyright Elsevier (1998). Numbers to the lower right represent anterior-posterior distance (mm) from bregma. Gray circles represent electrode placements. 8 actual recording sites were in the right and 3 in the left hemisphere. (**B**) Behavioral task design. See methods. d: day(s); LP_I_: Initiating lever press; LP_T_: Terminating lever press; Org 12.5% Suc: Orange-flavored 12.5% sucrose reward (training reward); Org 20% Suc: Orange-flavored 20% sucrose; Grp 12.5% Suc: Grape-flavored 12.5% sucrose; Poly: Polycose solution; Pellet; grain-based food pellet; H_2_O: water.

Fast-scan cyclic voltammetry (FSCV) was used to measure dopamine concentration changes in the NAc (Fig. 1A) of food-deprived rats throughout the acquisition and performance of a fixed sequence of two different lever-press actions to earn a sucrose reward (Fig. 1B). Chronically-implanted, carbon-fiber microelectrodes, which allow stable FSCV dopamine recordings from the same sampling space over months^19^, were employed to track longitudinal, within-subject changes in dopamine over the course of training and test. The control of self-initiated behavior is particularly disrupted by alterations in striatal dopamine signaling in Parkinson’s disease^20^ and addiction^21^, so we focused on a self-paced (*i.e.,* free-operant) action-sequence task devoid of experimenter-provided initiation cues. Subjects controlled both the initiation of each sequence and the speed with which it was performed. The initiating lever was continuously available and when pressed resulted in the insertion of the terminating lever into the chamber. The terminating lever remained out until it was pressed, at which point 0.1 ml of an orange-flavored 12.5% sucrose solution reward was delivered and this lever was retracted. Levers were located on the wall opposite the food-delivery port to dissociate dopamine signals related to collection of the earned reward from those related to initial approach towards the instrumental manipulandum and action execution. Rats earned 30 total rewards/session.

**Figure 2:**
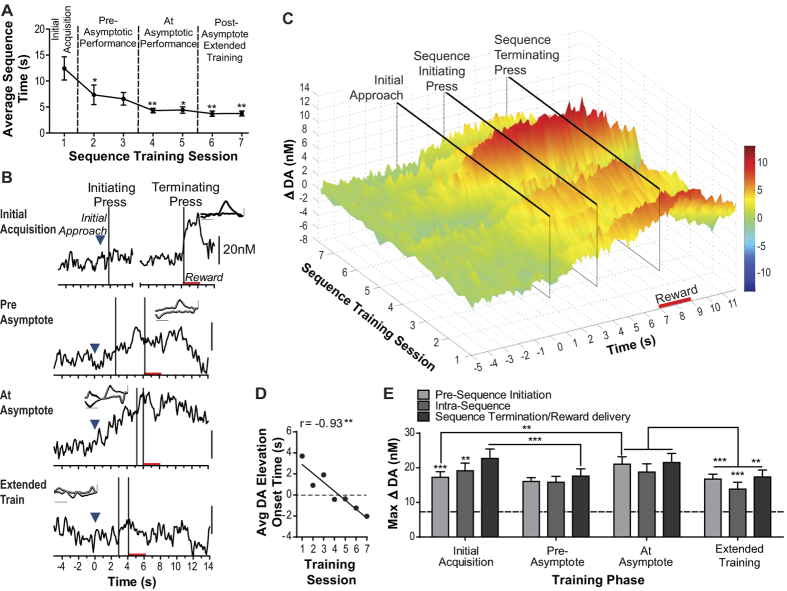
Dopamine concentration changes during action sequence learning and performance. (**A**) Average sequence completion time (initial lever press-reward collection) across training. (**B**) Single-trial representative dopamine concentration v. time traces from the same rat for each phase of training. All except the top trace where the break is indicated, show continuous, unaveraged, non-concatenated traces. Blue arrows: time of initial approach towards the initiating lever. Insertion of the terminating lever occurs immediately following the initiating lever press. Red bars: reward delivery and consumption (rats collected earned reward on average 0.27 s, SEM = 0.02, following the terminating lever press). Insets: Background-subtracted cyclic voltammograms (CVs) showing oxidation and reduction peaks that identify the detected electrochemical signal as dopamine. Initial acquisition CV from the peak of current following reward delivery. All other CVs are average of CVs taken at 1-s intervals for the duration of the concentration ramp starting 1 s (pre-asymptote) or 6 s (at asymptote and extended train) prior to the initiating press and extending 9 s after this press. Shading reflects +1 within-sample SEM. *X*-axis scale bar: 0.5 V and the *y*-axis scale bar: 0.5 nA. (**C**) Concatenated, baseline-subtracted dopamine concentration v. time traces (see Methods) were averaged across bins of 10 sequence completions (3 bins/training session), for each rat and then averaged across rats. Dopamine concentration change coded in false color. (**D**) Average dopamine concentration elevation onset time v. training session number within-subject correlation. (**E**) Trial-averaged peak dopamine concentration change during sequence execution (preceding the initiating press, after the initial press but prior to the terminating press, or following sequence termination when the reward was delivered and consumed). Dashed line: baseline root mean square (RMS) noise in the dopamine concentration trace in the absence of lever pressing activity. Error bars indicate +1 SEM. *p < 0.05, **p < 0.01, ***p < 0.001. See also Supplementary Figures 1–4 and Supplementary Table 1.

Rats’ behavior improved over the 7 days of action-sequence training (F_6,60_ = 8.52, *p* = 0.001; Fig. 2A). Asymptotic performance was, on average, reached on the 4^th^ training session, from which point on sequence completion time was lower than the first day of training (*p* < 0.05–0.01) and improved no further (*p* > 0.05). The first day of sequence training was, therefore, considered initial acquisition, with training sessions 2 and 3 considered ‘pre-asymptotic performance’, 4 and 5 considered ‘at asymptotic performance’, and 6 and 7 considered ‘extended training’ beyond asymptotic performance. With training, rats also began to re-initiate the next action sequence after earned reward collection more quickly (F_6,60_ = 8.77, *p* = 0.0008; re-initiation latency range: 45.50 s, SEM = 8.42, first session to 22.24 s, SEM = 3.17, last session).

### Dopamine signaling during action sequence learning and performance

As is apparent in the single-trial, representative examples (Fig. 2B) and group-averaged data (Fig. 2C), prolonged dopamine concentration changes were detected during sequence performance, the onset of which shifted as rats mastered the task. Voltammetric data were analyzed around 3 sequence event trigger points: the earliest identifiable approach towards the initiating lever, the execution of the initiating action, which was immediately followed by the insertion of the terminating lever into the chamber, and the execution of the terminating action that delivered the reward. The earned reward was collected within 0.3 s following the terminating press (first session average: 0.24 s, SEM = 0.04; last session average: 0.29 s, SEM = 0.07), and this latency did not change with training (F_6,60_ = 0.43, *p* = 0.77). The estimated time of the initial approach was identified in video recordings synchronized to the voltammetric data scored to determine when prior to the press the lever came into the rat’s sight line and then reversing the tape to identify the earliest movement on this path. Because the self-paced task allowed variable sequence times, dopamine concentration v. time traces were concatenated by scaling the peri-event windows to the lowest mean inter-event interval between consecutive events (initial approach to initiating press: 2.97 s, SEM = 0.77; initiating to terminating press: 4.03 s, 0.42) and plotting the data from each event to half of the inter-event interval, similar to the method described previously^11^. These traces were then trial-averaged. Representative example data (Fig. 2B and Supplementary Figure 1A–C) show evidence of the prolonged signaling profile in continuous (unaveraged, non-concatenated), single-trial dopamine concentration v. time traces, including those with longer inter-press intervals. Averaged current v. applied potential cyclic voltammograms (CVs) in examples provide evidence of the dopamine signature throughout the prolonged concentration change. Prolonged concentration elevations were not detected in the current component identified as pH, or in the residual unknown measurement component of the voltammetric data (Supplementary Figure 2).

In many cases, we identified a ‘ramping’ characteristic in the dopamine signal during action sequence execution, similar to that detected previously during spatial navigation^11^. Trial-averaged dopamine traces were identified as ramping if they exhibited a significant positive or negative linear regression coefficient (Pearson’s r, significant to at least *p* < 0.01) over the entire action sequence period, as described previously^11^. At asymptotic performance a significant (*p* < 0.01) linear regression coefficient was detected in 10/11 rats (r_170_ = −0.60–0.85), which was positive for the majority (7/11) of rats (see Supplementary Figure 1D and E for minority profile). The positive ramping characteristic was also detected at asymptotic performance in the majority (67.78%, SEM = 9.88) of individual trial dopamine concentration v. time traces for rats that displayed this profile in the trial-averaged trace. The prolonged ramping characteristic detected in the trial-averaged data was, therefore, not an artifact of averaging across transient dopamine elevations occurring at variable time points across trials. Regression coefficients for each training phase are presented in Supplementary Table 1. The positive ramping profile was not detected in the absence of lever-pressing activity. At asymptotic performance the majority of rats (9/11) actually showed a significant *negative* linear regression coefficient in the dopamine concentration v. time trace during the baseline period prior to the session onset, suggesting that these prolonged dopamine elevations did not emerge by chance, or by drift at the electrode (see Supplementary Figure 3).

NAc dopamine concentration began to rise increasingly earlier in the execution of the action sequence with training (Fig. 2C). There was a main effect of Training on dopamine elevation onset time (time of elevation >10% of peak concentration, relative to time of approach to the initiating lever; F_4,36_ = 3.83, *p* = 0.01), and a significant negative correlation between average dopamine elevation onset time and training session (r_7_ = −0.93, *p* = 0.002; Fig. 2D). At asymptotic performance dopamine concentration began to rise on average (though not on all trials for all rats, see representative examples) 2.03 s (SEM = 0.60) prior to the initial approach towards the initiating lever. The earlier onset time of the dopamine elevation with learning could be a secondary consequence of the overall elevation in its magnitude at asymptotic performance, but this was controlled for by defining onset time relative to concentration change magnitude. Moreover, when dopamine response magnitudes were equivalent (during initial acquisition and asymptotic performance) elevation onset time was still significantly earlier in sequence execution following training (t_10_ = 3.85, *p* = 0.003).

The dopamine response peak time also appeared to shift with training. During initial acquisition dopamine peaked during reward delivery/consumption following sequence termination, but with training the peak occurred earlier in the execution of the action sequence and at asymptotic performance occurred prior to execution of the initiating action. These observations were confirmed by statistical analysis of the maximal dopamine concentration change during sequence execution across training epochs (averaged across sequence-completion trials for each rat for each event: preceding the initiating press, after the initiating press, but before the terminating presses, or during reward delivery; Fig. 2E), which detected a significant effect of both Sequence event (F_2,20_ = 5.40, *p* = 0.01) and of Training (F_3,30_ = 3.89, *p* = 0.02), and an interaction between these factors (F_6,60_ = 2.81, *p* = 0.02). Post-hoc analyses (see Fig. 2E) further verified the observations described above. Although dopamine peaked earlier in sequence execution as rats reached asymptotic performance, it remained elevated until after the earned reward was collected, at which point it declined back to baseline before the next sequence was initiated. The dopamine response to the earned reward at asymptotic performance did not significantly differ from that induced by unexpected, non-contingent delivery of the same reward (t_10_ = 1.70, *p* = 0.12; Supplementary Figure 4). In no case were there any significant within-session changes in action sequence-related dopamine signaling; there was neither a main effect of Trial block (F_2,20_ = 0.005–2.36 *p* = 0.99–0.12) nor Trial block × Session phase interaction (F_2,20_ = 0.036–1.74 *p* = 0.84–0.16) on dopamine signaling during any training session.

### Dopamine correlates of acquisition and performance

**Figure 3:**
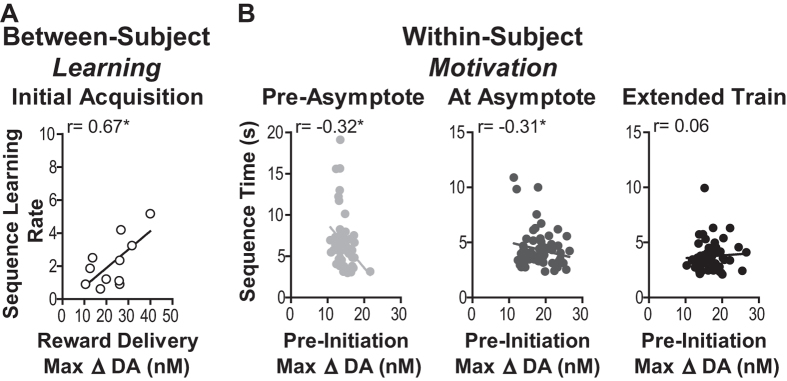
Dopamine correlates of acquisition and performance. (**A**) Peak amplitude dopamine response to the unexpectedly earned reward during the first sequence training session (averaged across earned rewards for each rat) v. rate of sequence task acquisition (slope to asymptotic performance) between-subject correlation. (**B**) Peak amplitude dopamine concentration change within 4 s prior to the initiating press v. time to complete the immediately following sequence within-subject correlation. All 30 sequence completions from each of the two training sessions/phase are included, with session number used as a controlling factor. *p < 0.05.

NAc dopamine concentration changes were found to correlate with task performance in several interesting ways. First, the trial-averaged peak dopamine response to reward delivery during initial acquisition positively correlated with future learning rate (between-subject correlation, r_11_ = 0.67, *p* = 0.02; Fig. 3A), such that rats for which the unexpectedly-earned reward during initial acquisition induced a larger amplitude dopamine concentration change subsequently learned the action sequence more quickly than those for which this response was smaller. Because NAc dopamine was shown to rise prior to sequence execution, we reasoned that it might also be related to the online motivation to engage in the task. In support of this, both prior to and at asymptotic performance the amplitude of dopamine elevation prior to sequence initiation significantly negatively correlated with time to complete the immediately following sequence (within-subject correlation, pre-asymptote: r_57_ = −0.33, *p* = 0.01, at asymptote: r_57_ = −0.31, *p* = 0.02, both controlling for session; Fig. 3B). Prior to and at asymptotic performance faster sequence trials tended to be preceded by larger amplitude dopamine elevations.

### Dopamine signaling during extended training

After extended training peak dopamine concentration amplitude preceding sequence initiation did not significantly correlate with sequence speed (r_57_ = 0.06, *p* = 0.65, controlling for session; Fig. 3B-right). This degradation in the correlational relationship corresponded to an apparent decoupling of dopamine from sequence performance with overtraining (see Fig. 2B–D). Indeed, the dopamine concentration elevation during sequence execution was significantly lower during extended training relative to levels at asymptotic performance (*p* < 0.01–0.001, Fig. 2E). Behavioral performance (*i.e.,* sequence speed) did not significantly differ between these sessions, ruling out this movement variable as an explanation for the disappearance of the dopamine response with extended training. These data suggest that NAc dopamine became unrelated to reward delivery and individual action sequence performance during extended training.

**Figure 4:**
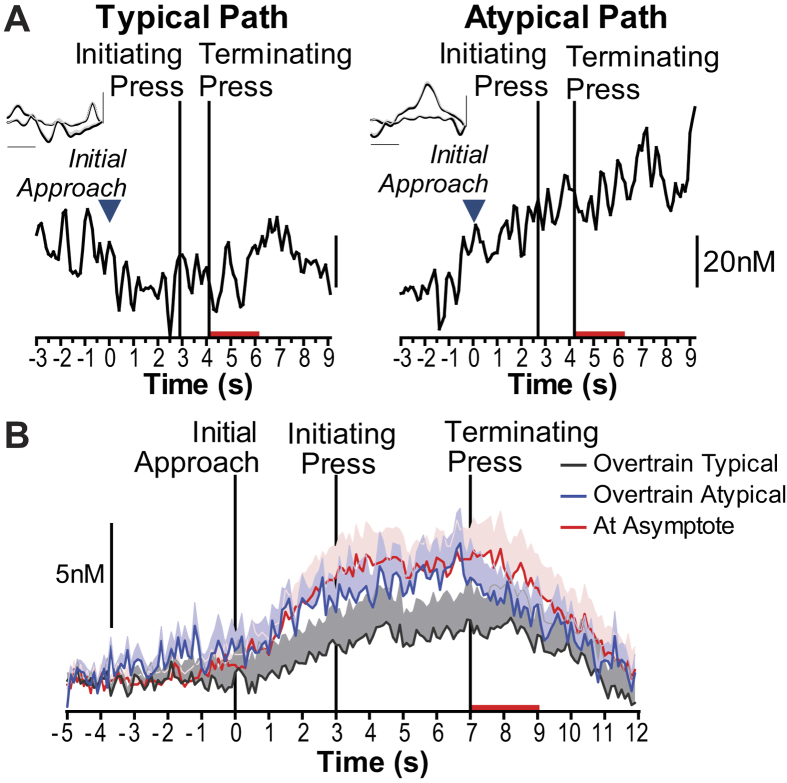
Dopamine signals following extended training during stereotyped v. atypical sequence paths. (**A**) Representative example of continuous (unaveraged, nonconcatenated) dopamine concentration v. time traces for individual sequence performance from the last training session from the same rat for a stereotypical and atypical sequence path trial. Blue arrows: time of initial approach towards initiating lever. Insertion of the terminating lever occurs immediately following the initiating lever press. Red bars: time of reward delivery and consumption. Insets: Averaged CVs taken at 1-s intervals starting 2 s prior to the initiating press and extending 7 s after this press; shading reflects +1 within-sample SEM; *x*-axis scale bar: 0.5 V, *y*-axis scale bar: 0.5 nA. (**B**) Average dopamine concentration v. time trace during action sequence performance at asymptotic performance and during overtraining divided for typical v. atypical path sequences. Shading reflects between-subject SEM.

Performance during extended training became largely stereotyped, but there were rare instances in which rats deviated from this stereotyped path, paused, or otherwise became disengaged in the task. We took advantage of these atypical trials to ask whether NAc dopamine signaling would be differentially engaged when rats reengaged in the task. Following reward collection, rats could to turn to the left or right upon exiting the food-delivery port to move to the opposite wall to reinitiate another sequence. By extended training rats developed a stereotypical path (path chosen >70% of the time). Atypical path trials (26.83% of trials during extended training, SEM = 2.82) occurred when rats deviated from their stereotyped path (e.g., exiting and turning to the left rather than typical right), paused on the stereotyped path before action execution, or otherwise became disengaged in the task. As can be seen in the representative examples (Fig. 4A) and group-averaged data (Fig. 4B) if rats deviated from their stereotyped action sequence path during extended training dopamine concentration was again elevated as rats executed the sequence. There was a significant main effect of Time (dopamine concentration averaged over 1-s bins for the entire sequence epoch and then trial-averaged for each subject; F_16,144_ = 7.83, *p* < 0.0001), with no effect of Type of Path (At Asymptote v Extended training- Typical v. Extended training- Atypical; F_2,18_ = 7.83, *p* < 0.0001), but a significant interaction between these factors (F_32,288_ = 1.84, p = 0.005). Although dopamine was not significantly elevated above baseline when rats executed the sequence on the overtrained stereotyped path (no main effect of time in action sequence on dopamine concentration: F_16,144_ = 2.32, *p* = 0.12), it was significantly elevated above baseline if they deviated from this path (F_16,144_ = 6.36, p = 0.002). Moreover, from the time of initial approach through reward delivery following sequence termination dopamine concentration was significantly lower than that elicited at asymptotic performance if the action was the overtrained stereotypical path (*p* < 0.05–0.001), but not if it was the overtrained atypical path (*p* > 0.05).

### NAc dopamine response to the most distal unexpected predictor of reward

**Figure 5:**
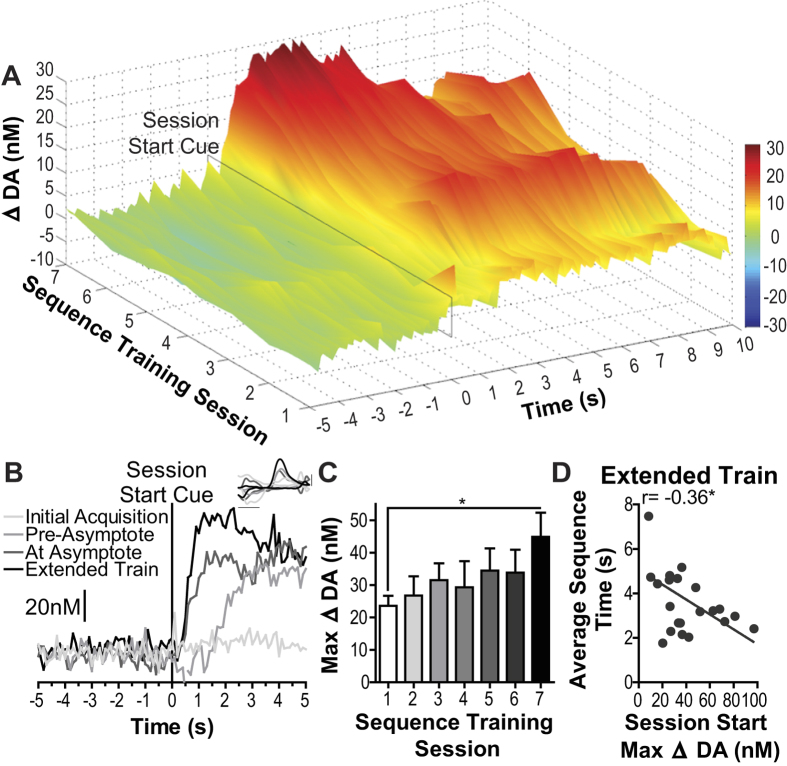
NAc dopamine response to the most distal predictor of reward. (**A**) Average baseline-subtracted dopamine concentration v. time traces surrounding presentation of the session start stimulus (light on and lever extended) for each training session. Dopamine concentration change coded in false color. (**B**) Representative dopamine concentration v. time trace around the session start cue from the same rat for each phase of training. Inset: background-subtracted CVs from the peak of current for each trace; *x*-axis scale bar: 0.5 V, *y-*axis scale bar: 1 nA. (**C**) Peak dopamine response to session start cue. (**D**) Peak amplitude dopamine response to session start cue v. average sequence completion time during the extended training phase between-subject correlation. Error bars indicate +1 SEM. *p < 0.05.

Because NAc dopamine signaling became decoupled from individual, stereotyped sequence performance after extended training, we next asked if this signal transitioned to an earlier task element. We evaluated dopamine signaling surrounding the most distal and unpredictable, task cue- that signaling the start of the session (house light turned on and lever extended into the chamber, occurring at a variable time ~20 min after being placed in the chamber). Secondarily, this analysis provided an opportunity to verify dopamine detection throughout training. The magnitude of dopamine response to this start cue was minimal early in training and became maximal on the last training session (group average Fig. 5A; representative example Fig. 5B; significant main effect of Training: F_4,38_ = 4.35, *p* = 0.006 on peak dopamine concentration, Fig. 5C). These data suggest that the dopamine response backpropagated to the most distal predictor of reward with extended training.

The magnitude of this cue-induced dopamine elevation during extended training significantly correlated with average sequence time (r_19_ = −0.36, *p* = 0.02, between-subjects, controlling for session; Fig. 5D), such that rats for which the session start cue elicited a larger amplitude dopamine response completed each sequence, on average, more quickly than those for which this elevation was smaller. The maximal magnitude of this cue-evoked dopamine response was larger than that detected during earlier training phases to individual reward delivery or sequence execution and that detected in response to non-contingent, unexpected reward delivery (Supplementary Figure 4). Though non-contingent reward delivery may have been somewhat expected because rats often sat in very close proximity to the food-delivery port during these tests.

### Dopamine signaling during expectation violation

After extended training dopamine concentration was robustly and transiently elevated by the most distal, unexpected cue predicting reward, but prolonged dopamine concentration changes were no longer detected during stereotyped action sequence performance. We next asked whether dopamine signaling would reemerge if the reward expectation was violated. After the sequence task was well learned, rats were given a series of tests in which they were allowed to perform the action sequence, but earned a reward different from the orange-flavored 12.5% sucrose expected based on training (see Fig. 1B). Two different types of expectation violations were conducted: value and identity. For the value expectation violation test the earned reward was substituted for one that was higher in value, but similar in specific identifying features (orange-flavored 20% sucrose). To determine if ramping dopamine signals would respond to a reward expectation violation absent a value shift, after extended training subsets of rats received a test in which the earned reward was substituted for one roughly similar in value, but different to varying extents in specific identifying features (e.g., taste, texture). These included a reward different in flavor only (grape-flavored 12.5% sucrose), an alternate liquid of equal caloric content (unflavored 12.5% polycose), an alternate food reward (grain food pellet) and a non-food reward (water, when water deprived). Each rat was given 3 of the 5 possible tests in randomized order and rats were allowed to earn 15 total rewards during each test. Preference for the higher value reward, but not any of the alternate identity rewards was observed in the rats used for the FSCV study during pre-training consumption of each reward in isolated homecage exposures (Supplementary Table 2). This also ensured that each violation reward was not novel at test.

**Figure 6:**
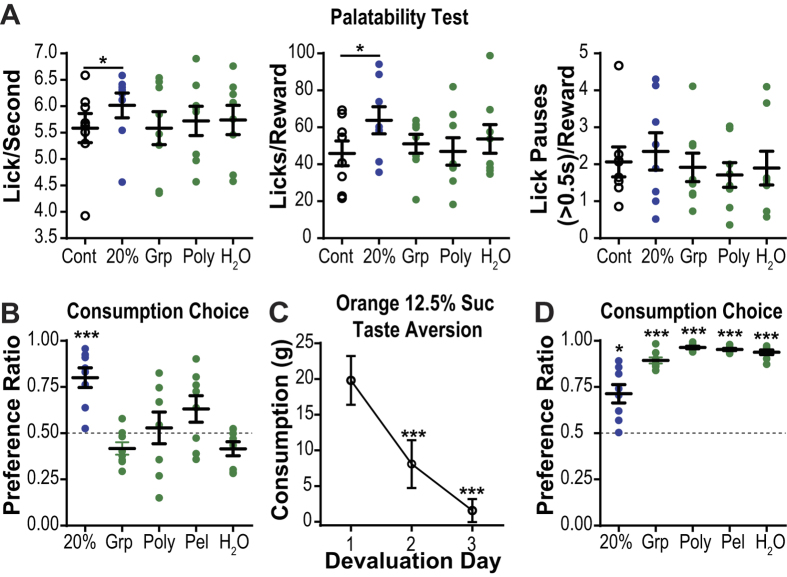
Reward preference and discrimination tests. Reward preference and discriminability tests were conducted in a separate group of rats from those used in the main FSCV study. Control reward: Cont, orange-flavored 12.5% sucrose. Higher value reward: 20%, orange-flavored 20% sucrose. Alternate identity rewards: Grp: Grape-flavored 12.5% sucrose reward; Poly: Polycose solution; Pel; grain-based food pellet; H_2_O: water. (**A**) Palatability test; isolated, non-contingent deliveries of each reward delivered in separate test sessions. Because this test requires a liquid reward it was not conducted for the pellet outcome Left: Lick frequency (Licks/second). *Y* axis truncated at floor lick rate of 3.5 licks/s based. Middle: Average number of licks/reward delivery. Right: Average number of pauses >0.5 s in licking/reward delivery. (**B**) Preference ratio [alternate reward consumption/(alternate reward consumption +12.5% orange sucrose consumption)] during consumption choice tests. (**D**) Consumption of the orange-flavored 12.5% sucrose on each of the 3 days it was followed by LiCl-induced nausea. (**E**) Preference ratios during consumption choice tests following conditioning of taste aversion to orange-flavored 12.5% sucrose. Errors bars indicate +1 SEM. *p < 0.05; ***p < 0.001. See also Supplementary Table 2.

To further test the value of each alternate reward relative to the orange-flavored 12.5% sucrose (that was used to condition the action sequence for subjects in the main FSCV experiment) we performed a series of preference tests in a separate group of rats. In the first test the relative palatability of each reward was evaluated by measuring lick frequency, total licks and pauses in licking (lasting > 0.5 s) during isolated, non-contingent deliveries of each reward in 30, 0.1 ml increments, during separate daily sessions^22^,^23^,^24^ (Fig. 6A). In the second, the relative preference for each reward was tested in a consumption choice test in which each violation reward was pitted against the orange-flavored 12.5% sucrose (Fig. 6B), as has been used previously to verify relative value^25^. In this test preference ratio in consumption served as a proxy measure of free choice. In both tests, the higher concentration sucrose solution was confirmed as a higher value reward than the orange-flavored 12.5% sucrose. Rats consumed more of the 20% sucrose solution over the 12.5% sucrose in the choice test (t_7_ = 4.48, *p* = 0.003; Fig. 5B). The 20% sucrose was also found to be more palatable, as reflected by consummatory licking reactions (Lick frequency: t_7_ = 3.43, *p* = 0.01; Licks/reward: t_7_ = 3.05, *p* = 0.02; Fig. 5A), though there were no differences in number of pauses in licking activity between the 12.5% and 20% sucrose (t_7_ = 0.54, *p* = 0.60; Fig. 5A- right). Each alternate identity reward was shown to be similar in value to the training outcome. In each case, there was no significant difference in consumption during the choice tests between the alternate identity reward and the orange-flavored 12.5% sucrose (*p* > 0.05, in all cases; no overall preference effect: F_1,7_ = 0.37, *p* = 0.56; Fig. 5B). The palatability of each alternate identity liquid outcome was also similar to the orange-flavored 12.5% sucrose (no main effect of preference on Lick frequency: F_3,21_ = 0.13, *p* = 0.91, Licks/reward: F_3,21_ = 0.38, *p* = 0.63, Licks/reward: F_3,21_ = 0.14, *p* = 0.91; post-hoc: *p* > 0.05 in all cases; Fig. 5A).

In this separate group of subjects, we also verified that each alternate identity reward was discriminable from the orange-flavored 12.5% sucrose. We conditioned a taste aversion to the orange-flavored 12.5% sucrose, by pairing with LiCl-induced nausea, and then repeated the consumption choice tests. The taste aversion treatment was effective; rats consumed significantly less of the orange-flavored 12.5% sucrose after it was paired with illness (F_2,11_ = 95.04, *p* < 0.0001; Fig. 6C). Although prior to conditioning the aversion to the orange-flavored 12.5% sucrose rats showed no significant preference between reward types, following this treatment rats consumed significantly more of each alternate identity reward than the now aversive orange-flavored 12.5% sucrose (F_3,21_ = 18.00, *p* < 0.0001; post hoc *p* < 0.0001, in all cases; Fig. 6D), indicating their discriminability. Rats also consumed more of the orange-flavored 20% sucrose relative to the now aversive orange-flavored 12.5% sucrose (t_7_ = 3.19, *p* = 0.02), but following the taste aversion rats did consume less of this higher value reward, even though it had not itself been paired with illness (t_7_ = 6.24, *p* = 0.0004), indicating that it shared many specific identifying features with the orange-flavored 12.5% sucrose.

**Figure 7:**
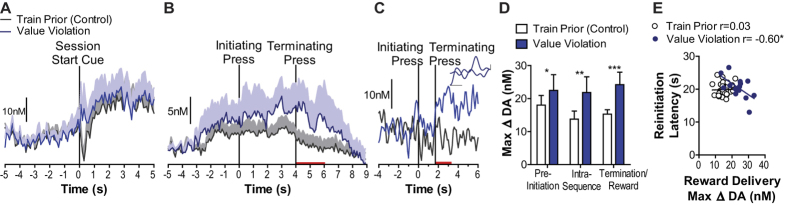
Dopamine signals during reward value expectation violation. (**A**) Average baseline-subtracted dopamine concentration v. time traces surrounding presentation of the session start stimulus for the preceding control training session (gray) and the test at which the value of the reward was unexpectedly increased (blue). Shading reflects +1 SEM. (**B**) Average dopamine concentration v. time trace during action sequence performance for the control training session and value expectation violation test. (**C**) Single-trial representative (continuous, unaveraged, nonconcatenated) dopamine concentration v. time traces from the same rat during sequence performance after extended training and during value expectation violation. Taken from the 9^th^ sequence completion trial. Red bars: time of reward delivery/consumption. Inset: background-subtracted CV from the peak of current for the test trace; *X*-axis scale bar: 0.5 V, *y*-axis scale bar: 0.1 nA. (**D**) Peak dopamine concentration change during sequence execution. (**E**) Trial-averaged peak amplitude dopamine concentration change to reward delivery v. latency to reinitiate the sequence following reward consumption (reward collection to next initiating press time) within-subjects correlation. Error bars indicate +1 SEM. *p < 0.05, **p < 0.01, ***p < 0.001. See also Supplementary Figure 5 and Supplementary Table 2.

For each expectation violation test in the FSCV experiment we first confirmed dopamine detection elicited by the session start stimulus. For the value expectation violation test this stimulus elicited a significant dopamine response and the magnitude of this response did not differ between test and the preceding training control session (main effect of Start stimulus: F_1,5_ = 15.93, *p* = 0.01, no effect of Value violation: F_1,5_ = 2.94, *p* = 0.15, or interaction: F_1,5_ = 0.42, *p* = 0.54; Fig. 7A). Importantly, as can be seen in the group-average shown in Fig. 7B and representative example shown in Fig. 7C, NAc dopamine concentration was elevated during sequence performance when the earned reward was unexpectedly higher in value, relative to the preceding control training session in which the rats’ reward expectation was not violated. There was a significant main effect of Reward value expectation violation on sequence execution dopamine responses (F_1,5_ = 6.52, *p* = 0.05), with neither an effect of Sequence event (F_2,10_ = 0.82, *p* = 0.47), nor a significant interaction between these factors (F_1,5_ = 2.26, *p* = 0.16; Fig. 7D). Notably, dopamine was elevated first when the unexpectedly higher value reward was initially discovered and then became elevated around action execution as the test proceeded (Supplementary Figure 5). The magnitude of the dopamine response to the unexpectedly larger value reward predicted performance. On trials in which the unexpectedly larger reward elicited a larger amplitude dopamine response rats reinitiated the next sequence more quickly (within-subject correlation, r_14_ = −0.60, *p* = 0.02; Fig. 7E). This was not the case for the preceding control session (r_29_ = −0.03, *p* = 0.89). Although there was evidence of sustained dopamine elevations during sequence execution, there was no clear evidence of the ramping characteristic in the dopamine signal during this value expectation violation test. The linear regression coefficient ranged from −0.51–0.68, with 33% of subjects showing a significantly positive slope and 50% showing a significantly negative slope on the trial-averaged dopamine concentration v. time trace.

**Figure 8:**
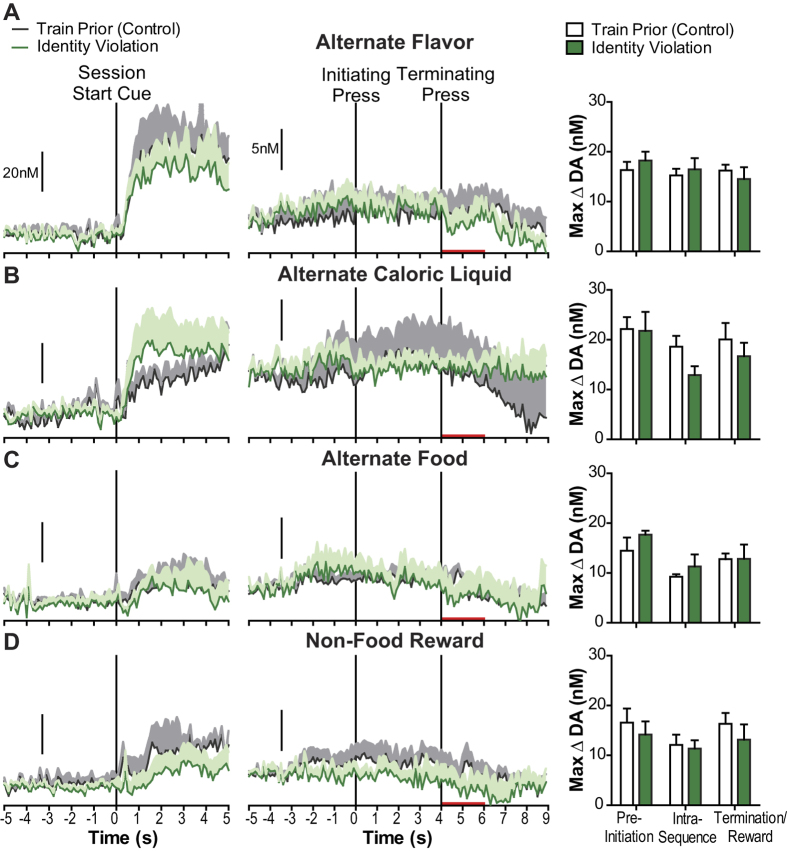
Dopamine signals during reward identity expectation violation. Left Panel: Average baseline-subtracted dopamine concentration v. time traces surrounding presentation of the session start stimulus for the preceding control training session (gray) and the tests at which the identity of the reward was altered (green). Shading reflects +1 SEM. Middle Panel: Average dopamine concentration v. time traces during action sequence performance for the control training session and identity expectation violation tests. Red bars: time of reward delivery and consumption. Right Panel: Trial-averaged peak dopamine concentration change during sequence execution. (**A**) Unexpected change in the flavor of the earned reward (grape-flavored 12.5% sucrose). (**B**) Unexpected change in the type of caloric liquid (12.5% polycose) (**C**). Unexpected change to an alternate food type (grain-based food pellet) (**D**). Unexpected change to a non-food reward (water when 18 hr water deprived). Error bars indicate +1 SEM. See also Supplementary Table 2.

In no case did NAc dopamine respond to an unexpected change in the identity, but not value of the earned reward. We first verified dopamine detection at the start stimulus. For each identity violation test the start stimulus induced a dopamine concentration elevation (Alternate Flavor: F_1,3_ = 18.08, *p* = 0.02, Fig. 8A- left; Alternate caloric liquid: F_1,4_ = 10.54, *p* = 0.03, Fig. 8B- left; Alternate food: F_1,3_ = 12.38, *p* = 0.04, Fig. 8C- left; Non-food reward: F_1,4_ = 15.30, *p* = 0.02, Fig. 8D- left), and there was no significant difference in this elevation between the violation test and the control training session (no main effect of violation, Alternate Flavor: F_1,3_ = 0.36, *p* = 0.59; Alternate caloric liquid: F_1,4_ = 0.04, *p* = 0.85; Alternate food: F_1,3_ = 0.79, *p* = 0.44; Non-food reward: F_1,4_ = 5.38, *p* = 0.08). Apparent differences in the magnitude of the start cue-evoked dopamine are due to between-subject variability in this response. In no case did the magnitude of the start cue dopamine response at test differ from that induced on the last training session day (Alternate Flavor: t_3_ = 0.08, *p* = 0.94; Alternate caloric liquid: t_3_ = 0.005, *p* = 0.997; Alternate food: t_3_ = 1.92, *p* = 0.15; Non-food reward: t_3_ = 1.50, *p* = 0.21). Importantly, sequence execution-related dopamine signaling did not differ between any of the identity violation tests and their preceding control training session (Fig. 8- middle). There was no main effect of Identity expectation violation on sequence action- or reward-related dopamine concentration when only the flavor of the reward was switched (F_1,3_ = 0.04, *p* = 0.84; Fig. 8A- right), when the reward was an unexpectedly different caloric solution (F_1,4_ = 1.80, *p* = 0.25; Fig. 8B- right), when the reward was switched to an entirely different food (F_1,3_ = 0.42, *p* = 0.56; Fig. 8C- right), or when the reward was switched to non-food appetitive item (water when water deprived; F_1,3_ = 0.59, *p* = 0.49; Fig. 8D- right). In all cases there was no evidence of a significant dopamine concentration elevation above baseline during action sequence execution, despite the reliable detection of dopamine to the session start stimulus. Because there was some variability in the preference ratios for the alternate rewards (especially polycose and food pellets) relative to the orange-flavored 12.5% sucrose reward (see Fig. 6B), we looked for a relationship between subjective preference for the alternate identity reward (as measured by relative consumption in isolated home cage exposures) and task-related dopamine signaling. These analyses are presented in Supplementary Table 2 and showed no evidence of any significant relationship. These data, therefore, suggest that an unexpected change in the identity of the reward was not sufficient to reengage NAc dopamine signaling. Behavioral effects of the expectation violation are provided in Supplementary Table 2.

## Discussion

We used FSCV to monitor NAc dopamine concentration changes throughout the acquisition and performance of a self-paced, instrumental action sequence. Prolonged dopamine concentration changes were detected at asymptotic performance that gradually increased as rats executed the sequence and decreased following collection of the earned reward. The prolonged signaling profile detected here contrasts to the transient signals that have been detected previously during the execution of single cued^26^,^27^,^28^,^29^,^30^,^31^,^32^, or self-initiated^26^,^33^,^34^,^35^ actions. Clear evidence of prolonged dopamine elevations was identified in the unaveraged, non-concatenated, single-trial data, suggesting that this profile was not result of time averaging across punctate dopamine changes occurring at variable time points around action execution. These prolonged NAc dopamine signals did, however, match the profile (both duration on the order of seconds and magnitude) of signals previously detected, and found to encode spatial, but not temporal proximity to reward, in the ventral striatum during performance of a reward-seeking maze^11^. This prolonged form of NAc dopamine signaling is, therefore, not exclusively engaged by spatial navigation across long distances to earn reward, but also occurs within a more limited spatial environment when a sequence of ordered actions is required to obtain reward. However, we note that unmonitored spatial navigation factors (e.g., distance to initiating lever, latency to motion onset, etc.) likely contributed to the signaling profile detected here.

We previously reported similarly sustained NAc dopamine concentrations changes during action sequence execution in pre-trained rats^7^; the longitudinal within-subject data here revealed how such signaling is dynamic with learning. Initially the unexpected earned reward elicited a large dopamine response, but with task acquisition prolonged dopamine signals emerged and began to rise increasingly earlier in the execution of the action sequence with training. This concords with the previously reported differences in NAc dopamine signaling in untrained v. pre-trained rats^7^. Here we found that dopamine elevations also became larger in magnitude when behavior improved to asymptotic levels relative to pre-asymptotic performance, a finding in line with data showing greater reward cue-induced dopamine cell activity with faster cued response times^36^. Interestingly, at asymptotic performance the NAc dopamine elevation began prior to the rats’ own unprompted initiation of the sequence, as has been shown previously in both dopamine cell activity^37^,^38^ and NAc release^7^,^33^,^34^. This suggests dopamine signaling may be triggered by some internal or other stimulus known only to the behaving subject important for initiating action and is consistent with findings that dopamine receptor activation facilitates the excitation of NAc medium spiny neurons^39^, the activity of which encodes locomotor onset latency^40^.

Dopamine signaling was further dynamic with extended training, actually becoming decoupled from stereotyped action sequence execution. This accords with findings that NAc dopamine responses to predictable Pavlovian cues^41^ and instrumental actions^7^,^35^ are attenuated with overtraining. Though we cannot conclusively say that performance following overtraining in our, or these previous tasks, was habitual, because neither the response-outcome contingency, nor outcome value were manipulated, these results are consistent with those demonstrating that overtrained habitual behaviors are less dependent on (though not entirely independent of) NAc dopamine receptor signaling^42^.

This finding is, however, inconsistent with the prolonged NAc dopamine signals detected during spatial navigation in presumably well-trained rats^11^. This discrepancy may be the result of the task differences (lever press response v. maze run), or the difference in sampling locations (NAc core v. ventromedial striatum). It is, perhaps, more likely that it results from task predictability and response flexibility. In our task rats self-initiated each sequence trial and, therefore, could well predict all task events and respond stereotypically. The maze task in Howe *et al.* (2013)^11^ followed an experimenter-initiated trial structure in which rats had to navigate ‘on the fly’ based on experimenter-provided cues revealing reinforcement location. Prolonged dopamine signals may have persisted in this task due to this unpredictability and inability to stereotype behavior. Indeed, the ability to predict upcoming state changes has been shown to be critical to the diminution of task-related dopamine signaling with overtraining^41^. If after overtraining in the current experiment rats became disengaged in the task, prolonged dopamine concentration elevations reemerged when the rat flexibly reengaged in the task, corresponding to findings that NAc dopamine is required for ‘flexible’ approach to an instrumental operandum^17^.

Although NAc dopamine concentration was no longer elevated during stereotyped action sequence performance following overtraining, dopamine elevations were robustly elicited in response to the unexpected presentation of the most distal reward predictor, the session start stimulus. This backpropagation of the dopamine signal is consistent with recent findings of dopamine responses during performance of an action-sequence task largely similar to that used here, with the critical exception that it was on an experimenter-defined trial structure in which rats were unable to predict the time of the next sequence trial. Following post-asymptotic performance in this task, NAc core dopamine was transiently elevated by the unexpected presentation of the initiating lever^43^. That the maximal magnitude of the start stimulus-induced dopamine signal detected here was significantly larger than that of the dopamine concentration changes detected during asymptotic sequence execution perhaps reflects this stimulus’s higher value, because it signals the opportunity to earn many rewards, corresponding to findings that dopamine cell activity can encode the value of multiple future rewards^10^.

NAc dopamine signaling was found to be reengaged after overtraining if the earned reward was unexpectedly larger in value, but not if only its specific identity (e.g., taste) was altered. This suggests that NAc dopamine (at least in the form of prolonged signals) encodes information about the motivational value, rather than the specific sensory features of rewards. This interpretation is consistent with previous work demonstrating NAc dopamine responses to valuable sucrose reward in the absence of its identifying sweet taste^44^. These findings also accord the encoding of reward value^45^,^46^,^47^ by dopamine cell body firing, and with findings that the NAc is required to learn from an unexpected reward value change^48^.

The prolonged dopamine signals detected here during action sequence execution and previously^11^ are more protracted than typical phasic signaling, but rise and fall too quickly to be considered tonic changes. Although we did not fully test whether the observed dopamine signaling profile was or was not a reward-prediction error in the strict sense, we note several elements of the signal detected here could be interpreted as consistent with the well-supported role for *phasic* dopamine signaling in reporting the reward-prediction errors vital for associative encoding^1^,^2^,^3^,^4^. These include: 1. the robust dopamine response to the unexpectedly earned reward during initial acquisition, the amplitude of which predicted subsequent learning rate, 2. the backpropagation of dopamine elevations to the most distal and unexpected reward-predictive stimulus with extended training and 3. the reactivation of dopamine signaling by unexpected delivery of a higher value reward in fully-trained rats. Other elements of the dopamine signals detected here do not perfectly reflect prediction error encoding. Firstly, the re-emergence of prolonged dopamine elevations by the larger than expected reward could be due to the presence of positive prediction errors, but could also result from a unidirectional attention, alerting, or motivating function. Future work is required to clarify whether prolonged dopamine signaling can be altered by negative prediction errors, as has been identified for transient NAc dopamine release^6^,^43^,^49^ and cell body firing^1^,^3^,^50^,^51^,^52^. Secondly, at asymptotic performance dopamine concentration remained elevated during delivery and collection of the presumably expected earned reward. The persistent elevation of dopamine concentration to earned reward replicated our previous finding in a similar instrumental sequence task^7^ and the findings of prolonged dopamine elevations during spatial navigation^11^. Interestingly, several recent computational advances have been made to allow a temporal difference reinforcement learning model to predict a dopamine concentration ramp by considering either a non-linear representation of space^53^ (which may be specific to spatial navigation), or time-dependence of dopamine-mediated plasticity^54^.

Findings inconsistent with prediction error encoding by prolonged dopamine elevations do not suggest refutation of this theory, but rather highlight the possibility that there are multiple roles for dopamine, some over different time scales^49^,^55^. This has long been understood for the distinct modes of tonic and phasic signaling^55^,^56^. The bursting activity of dopamine cell bodies, which has been elegantly demonstrated to encode reward-prediction errors^1^,^2^,^3^, is thought to mediate phasic dopamine concentration changes^57^, but terminal dopamine does not always maintain fidelity with this cell body activity. Indeed, prolonged activity has not been demonstrated in dopamine firing during procurement of certain reward^58^. Modulatory mechanisms at striatal terminals can regulate the ‘decoding’ of dopamine cell firing into release and can even induce release independent of cell body activity^55^,^59^. Striatal cholinergic signaling provides one such mechanism^60^,^61^ and, interestingly, prolonged activity of ventral striatal interneurons (many of which are cholinergic) during reward-related behavior has been reported^62^.

As proposed by Howe *et al.* (2013), prolonged dopamine elevations may reflect the sustained motivational drive required to pursue distal rewards. In support of this here, dopamine elevations preceding sequence initiation predicted the speed of the immediately following sequence, corroborating our previous report^7^. These findings are also consistent with data suggesting that NAc dopamine encodes a dynamic, temporally-discounted, value estimation of future rewards important for motivating action^49^. Because it was not detected in the current data during stereotyped sequence execution, the prolonged form of dopamine signaling may be particularly important for motivation of flexible actions. The task here was ostensibly instrumental (action-reward), but Pavlovian (stimulus-reward) incentive processes likely exerted a strong influence over its performance. Internal proprioceptive signals, the sight of the initiating lever, and the terminal lever insertion were all cues signaling the opportunity for and occurrence of reward and, therefore, likely contributed to the prolonged dopamine elevations during sequence execution. Indeed, Pavlovian cues can evoke sustained dopamine signaling, elements of which correlate with the motivational influence of the cue over instrumental action^63^,^64^. NAc dopamine is also necessary for such Pavlovian incentive processes^18^,^65^,^66^,^67^,^68^,^69^,^70^,^71^. Furthermore, NAc dopamine signaling was not only re-engaged by an unexpectedly larger value reward, but its correlation to performance also reemerged. Like the dopamine response detected here, Pavlovian incentive motivation does not depend on specific reward identity^72^. Even when dopamine transitioned to the start cue, it predicted task performance. Entire sets of action sequences can become ‘chunked’ into a single behavioral unit^73^,^74^,^75^, such that NAc dopamine elevations to this most distal unexpected reward predictor could relate to hierarchical motivational control over a large chunk of stereotyped actions.

## Materials and Methods

### Subjects

Male, Sprague Dawley rats (n = 11; 280–320 g upon arrival; Charles River Laboratories, Wilmington, MA) were group housed and handled daily prior to surgery for 5–7 days. Rats were maintained on a food-deprived schedule whereby they received 10–12 g of their maintenance diet daily in order to maintain approximately 85% free-feeding body weight. Rats were provided free access to tap water in the home cage and were fed approximately 3–4 hr after each daily training session. All procedures were conducted in accordance with the NIH Guide for the Care and use of Laboratory Animals and approved by the UCLA Institutional Animal Care and Use Committee.

### Electrode preparation, calibration, and implantation

Carbon-fiber microelectrode preparation and calibration procedures are identical to those described previously^19^,^63^, and are detailed in the Supplementary Methods. The average calibration factor was 40.61 nM/nA (SEM = 1.79). Calibrated microelectrodes were chronically-implanted into the NAc- core region as described previously^63^. Recording locations were verified with histological procedures described previously^63^ (see also Supplementary Methods) and are presented in Fig. 1A.

### Behavioral task and voltammetry data acquisition

Training commenced between 30–36 days post-surgery (see Supplementary Methods for pre-training acclimation procedures). All training and testing took place in 2 Med Associates operant boxes (East Fairfield, VT) described in the Supplementary Methods. FSCV was used to measure dopamine fluctuations in NAc, as described previously^19^,^63^ and as detailed in the Supplementary Methods, throughout the acquisition of a self-initiated action sequence. For each session rats were placed in the operant chamber and tethered to the voltammetric recording unit. After stabilization of the baseline current (approximately. 20 min) in the dark chamber the behavioral session commenced with the onset of the house light and insertion of the lever as appropriate. The action-sequence behavioral task required rats to perform a fixed sequence of two different lever-press actions to earn the orange-flavored 12.5% sucrose reward. The ‘initiating’ lever was continuously available and when pressed resulted in the insertion of the ‘terminating’ lever into the chamber. Pressing the terminating lever resulted in sucrose delivery into a port located on the wall opposite the levers and caused this lever to be retracted. The task was entirely self-paced; there were no experimenter imposed response-time requirements or restrictions on sequence performance. Each training session was terminated when rats earned 30 total rewards. Subjects were trained for 7 total days.

To evaluate dopamine responses to violations in reward prediction, after the sequence task was well learned, 9 of the 11 rats were given a series of tests in which they were allowed to perform the action sequence, but earned a reward different from that expected based on training. Each rat received 3 of the 5 possible tests in randomized order, with the exception of 3 rats who were given only 2 tests due to an inability to make reliable recordings on the third test day. Rats were retrained with the original orange-flavored 12.5% sucrose outcome in between each test. Retraining performance did not significantly differ from performance on the last training session prior to test (No main effect of Retrain/violation F_4,19_ = 1.60, *p* = 0.22, Type of violation F_1,19_ = 0.10, *p* = 0.75, or interaction F_4,19_ = 1.60, *p* = 0.22 on average sequence completion time). An orange-flavored 20% sucrose reward was substituted (n = 6; 1 subject received this violation on Test 1, 3 on Test 2 and 2 on Test 3) for the reward value expectation violation. During a pre-training consumption test (15-min access to each reward on successive days) rats consumed more of this reward than the orange-flavored 12.5% sucrose training outcome (Supplementary Table 2). The remainder of the alternate rewards were all found to be equivalent in value (see tests below) to the training outcome, but differed to varying extents in their sensory-specific identity features. Grape-flavored 12.5% sucrose (n = 4; 3 Test 1, 1 Test 2) was used as a reward different in flavor only. Unflavored 12.5% polycose (n = 5; 2 Test 1, 3 Test 2) served as reward that was equal in caloric content, but of a different form of liquid carbohydrate and a distinct flavor. Previous work has demonstrated the roughly equal preference for and palatability of polycose to sucrose at the concentrations used here^76^. A 45 mg grain pellet (Bio-Serv, Frenchtown, NJ; n = 4; 2 Test 1, 1 Test 2, 1 Test 3) served as an alternate type and texture of food reward. Water (n = 5; 1 Test 1, 1 Test 2, 3 Test 3) served as a non-food reward. Rats were given full access to food, but deprived of water for 18 hr prior to the water test to ensure this reward was motivationally relevant. All other tests were conducted 18 hr food deprived as in training. Each test session was terminated after rats earned 15 total rewards.

### Reward preference and discrimination tests

A series of preference and discrimination tests were performed in a separate group of rats (n = 8; male, Sprague Dawley 280–320 g upon arrival; Charles River Laboratories, Wilmington, MA) to confirm the relative value and discriminability of each violation test reward against the orange-flavored 12.5% sucrose that served as the earned reward in the main experiment. Two preference tests were used. In the first, the relative preference for each reward was tested in consumption choice test wherein each violation reward was pitted against the orange-flavored 12.5% sucrose. On separate days rats were given 15-min access to the orange-flavored 12.5% sucrose along with one of the violation test rewards (orange-flavored 20% sucrose, grape-flavored 12.5% sucrose, unflavored 12.5% polycose, grain pellets, or water). All tests were conducted 18 hr food deprived, unless the alternate water reward was used, in which case rats were 0 hr food deprived, but 18 hr water deprived. In the second, test the relative palatability of each reward was evaluated by measuring lick frequency, total licks, and pauses of >0.5 s in licking during isolated non-contingent delivery of 0.1 ml of each reward (each reward delivered in separate test sessions) as described previously^22^. Because this test requires a liquid reward it was not conducted for the grain pellet outcome. To ensure all violation test rewards were discriminable from the orange-flavored 12.5% sucrose we conditioned an aversion to this reward by pairing with LiCl-induced nausea and then the consumption choice tests were repeated. Taste aversion treatment consisted of 3 days of 30-min access to the orange-flavored 12.5% sucrose followed by injection of LiCl (0.15 M LiCl, 20 mL/kg, i.p.). Consumption choice tests proceeded on the day following the last taste aversion conditioning session.

### Data analysis

Unless otherwise mentioned all data were processed with Microsoft Excel (Redmond, WA) and MATLAB (MathWorks, Inc., Natick, MA). Statistical analyses were conducted with GraphPad Prism (La Jolla, CA) and SPSS (IBM Corp, Chicago, IL). For all hypothesis tests, the α level for significance was set to *p* < 0.05. Data were analyzed with one- and two-way ANOVAs (Geisser-Greenhouse correction), paired t-tests, correlation and regression where appropriate. Post-hoc comparisons correcting for multiple comparisons were used to clarify all main effects and interactions.

#### Behavioral Analysis

Action-sequence time (time between the initiating lever press and reward collection) was the primary behavioral output measure. Re-initiation time (time from reward collection to the next initiating lever press) was used as a measure of the self-imposed inter-trial interval. The slope of the change in average sequence time from the first training session to asymptotic performance was the measure of learning rate.

Video recordings of all experiments were synchronized to behavioral and electrochemical data and were scored to identify the time at which rats began movement towards the initiating lever. Time of this ‘initial approach’ was estimated by determining when prior to the press the lever came into the rat’s sight line (horizontal line from nose) and then reversing the tape to identify the earliest movement on this path. Only those initiating movements that culminated in an actual lever press were included in this analysis.

Stereotyped v. non-stereotyped response paths during the extended training phase were also evaluated in these recordings. When rats earned a reward they could either turn to the left or to the right upon exiting the food-delivery port to make the initiating movement to the back of the box to reinitiate another sequence and were free to pause or otherwise deviate from the direct path to the lever. By extended training rats developed a stereotypical path, defined as the path that chosen >70% of the time (e.g., turning right out of the food-delivery port and proceeding directly to the initiating lever). An atypical path was defined as either the alternate to the typical path (e.g., leftward movement if the typical path was right) *or* a pause in movement or movement away from the lever on the typical path, such that when rats re-engaged in the sequence they did so from a different starting location. One rat was omitted from this analysis due to an inability to score typical v. atypical path.

#### Voltammetric Analysis

Electrochemical data were analyzed using software written in LabVIEW (National Instruments). Principal component regression (PCR), a chemometric technique that combines principal component analysis with inverse least-squares regression^77^,^78^, was used to isolate changes in current due to dopamine and pH^80^ from the cyclic voltammetric data. We used a standard training set of current versus applied potential (CV) templates for known dopamine and pH concentrations of varying concentrations *in vitro* from a sample of representative carbon fiber microelectrodes, as has been described previously^11^,^30^,^63^,^64^,^66^. CVs of *in vivo* background drift from these electrodes were also including in the training set, as previous work has dictated^78^. After this analysis all data were converted to an estimated dopamine concentration via an electrode-specific, *in vitro* pre-test calibration factor.

Voltammetric data were isolated and concatenated starting 5 s before the initial approach toward the initiating lever and ending 5 s after the delivery of the earned reward. Concatenation was performed by scaling the peri-event windows to the lowest mean inter-event interval between consecutive events (initial approach to initiating press latency: 2.97 s, SEM = 0.77; initiating to terminating press latency: 4.03 s, SEM = 0.42) and plotting the data from each event to half of the inter-event interval, similar to the method described previously^11^. This was also vital to allow comparison of results to aforementioned findings of prolonged dopamine concentration changes during spatial navigation^11^. For each sequence completion ‘trial’ voltammetry data were normalized by subtracting average background current measured during the 1-s baseline period 5 s prior to the initial approach. Within this time frame dopamine responses to reward delivery/collection could be distinguished from those related to the initiation of the next sequence; the average self-imposed inter-trial interval was 22.42 s (SEM = 3.03) at the final day of training. Linear regression analysis was conducted on the average dopamine concentration v. action-sequence time traces for each session for, each rat to determine the presence or absence of the ‘ramping’ characteristic, i.e., gradual increases or decreases during sequence execution, as described previously^11^. These traces were also used to determine the dopamine concentration elevation onset (point at which dopamine concentration became elevated above 10% of the peak concentration change). Linear regression analysis was also performed on each individual trial dopamine concentration v. time trace.

Average sequence time was always considerably less than the 90-s limit on PCR prediction of *in vivo* FSCV data^77^ (see Fig. 2A), preventing this concatenation from being significantly influenced by baseline drift^78^. Baseline subtraction ensured dopamine concentration estimates were relative to an immediately preceding baseline concentration. Individual values of residual extraneous variance in unknown measurement (*Q*) from the PCR analysis were compared to the average tolerance level (*Qα*), which in these measurements was 174.12 *Q*/nA^2^. On occasion isolated transient electrical artifacts were detected, as identified by transient *Q* values well in excess of the *Qα* in isolated cells. Estimates of dopamine at these time points were interpolated by taking the average of the surrounding cells. Trials in which >10% of the data points had residual values in excess of the *Qα* were omitted from the analysis. This occurred on <5% of all trials. All PCR residual results are provided in Supplementary Figure 3C and D.

Voltammetric data for the test phase were analyzed similarly with the exception that the initiating press, rather than initial approach served as the first analyzed event to focus on reward- and action-related dopamine concentration changes. Data were also analyzed for the period 5 s prior to and 10 s after the start of the session (house light on, lever out, occurring approximately ~ 20 min after the rat was placed in the dark operant chamber). Peak dopamine concentration change was quantified for each trial in each analysis window and this value was averaged across each training/test session for each rat. Root mean square (RMS) noise in similar time epochs outside of the behavioral sessions (e.g., in the absence of lever-pressing activity) are provided as a reference for this measure.

## Additional Information

**How to cite this article**: Collins, A. L. *et al.* Dynamic mesolimbic dopamine signaling during action sequence learning and expectation violation. *Sci. Rep.* **6**, 20231; doi: 10.1038/srep20231 (2016).

## Supplementary Material

Supplementary Information
