# Supplementary material for: Dynamic mesolimbic dopamine signaling during action sequence learning and expectation violation
Source: Sci Rep. 2016 Feb 12;6:20231. doi: 10.1038/srep20231 (PMC4751524; doi:10.1038/srep20231)
Supplement: Supplementary Information [file srep20231-s1.pdf]

Dynamic mesolimbic dopamine signaling during action sequence learning and expectation violation

Anne L. Collins[1], Venuz Y. Greenfield[1], Jeffrey K. Bye[1], Kay E. Linker[1], Alice S. Wang[1], Kate M. Wassum[1,2]

1. Dept. of Psychology, UCLA, Los Angeles, CA 90095. 2. Brain Research Institute, UCLA, Los Angeles, CA 90095, USA.

SUPPLEMENTARY MATERIALS AND METHODS

Electrode preparation and calibration.

A 7-μm diameter carbon fiber (Goodfellow Corporation, Coraopolis, PA) was encased within a 10-mm length of 90-μm diameter polyimide-covered fused-silica capillary (Polymicro Technologies, Phoenix, AZ) and sealed at the tip with epoxy allowing an exposed length of ~100-150 μm. All carbon-fiber microelectrodes were pre-calibrated with dopamine (0.25-1 μM in phosphate buffered saline, pH=7.4) in a custom-made flow cell (flow rate 4 ml/min) prior to implantation. The average calibration factor was 40.61 nM/nA (SEM=1.79). Preliminary pre- and post-implantation calibrations suggest this value changes <10% due to of chronic implantation for ~70 days.

Voltammetry data acquisition.

FSCV was used to measure dopamine concentration changes in NAc throughout the acquisition of a self-initiated action sequence. Recordings commenced between 30-36 days following surgery. For each session rats were placed in the operant chamber and tethered to the voltammetric recording unit through an electrical swivel (Crist Instrument Co, Hagerstown, MD). A custom-made voltammetric potentiostat was used to apply a triangular waveform to the carbon-fiber microelectrode through a head-mounted voltammetric amplifier. The applied potential was held at -0.4 V (vs. the Ag/AgCl reference) and then ramped to +1.3 V and back to -0.4 V at 400 V/s, repeating every 100 ms for a sample rate of 10 Hz. Waveform generation and resultant data acquisition were carried out using 2 PCI multi-function data acquisition cards and custom software written in LabVIEW (National Instruments, Austin, TX). After stabilization of the baseline current (approx. 20 min) in the dark chamber the behavioral session commenced with the onset of the house light and insertion of the lever as appropriate.

Behavioral apparatus.

All training and testing took place in 2 Med Associates operant boxes (East Fairfield, VT) that contained a recessed food-delivery port in the front wall and 2 retractable levers that could be inserted on either side of the wall opposite the food port. A photobeam entry detector was positioned at the entry to the food port. The chambers were also equipped with syringe pump to deliver solution, as described below, in 0.1 ml increments through a stainless steel tube into a well in the food port and a pellet dispenser to deliver single food pellets into the same port. A 3-watt, 24-volt house light mounted on the top of the back wall opposite the food cup provided illumination.

Behavioral pre-training.

Prior to training on the action-sequence task, rats received 3 sessions of non-contingent exposure to the orange-flavored 12.5% sucrose solution reward that would serve as the earned outcome (30, 0.1 ml/2 s outcomes over 40 min) in the operant chamber with the levers retracted. Rats were then given 1 day of single-action training on what would become the terminating lever prior to full action sequence training.

Histological verification of recording sites.

At the conclusion of each experiment rats were deeply anesthetized with Pentasol (100 mg/kg i.p.) and the recording site was marked by making a small electrolytic lesion at the electrode tip by passing a current (~70 μA) through the carbon fiber microelectrode for 20 s. Rats were then trans-cardially perfused with 0.9% saline followed by 10% formalin. The brains were removed and post-fixed in paraformaldehyde, then cryosectioned into 50 μm slices, mounted onto slides and stained with cresyl violet. Light microscopy was used to examine electrode placement in the NAc. Histological data are presented in Figure 1.

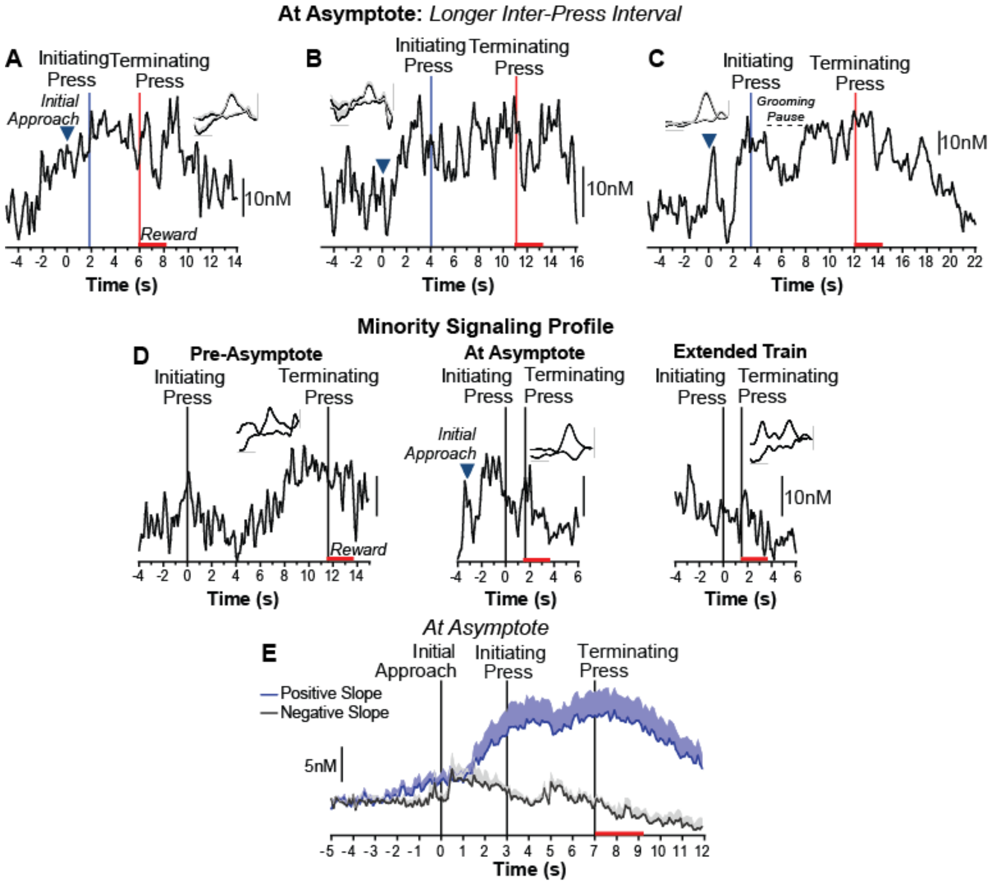

Supplementary Figure 1. Related to Figure 2. Dopamine signaling profiles at asymptotic performance. A-C. Single-trial representative continuous (unaveraged, nonconcatenated) dopamine concentration v. time traces during action sequence execution, each from a different subject. Blue arrows- time of initial approach towards initiating lever. Insertion of the terminating lever occurs immediately following the initiating lever press. Red bars- time of reward delivery and consumption. Insets: Average of CVs taken at 1-s intervals for the duration of the concentration elevation; shading reflects +1 within-sample SEM; *x*-axis scale bar indicates 0.5 V and the *y*-axis scale bar indicates 0.5 nA. **A & B**. Examples of continuous sequence performance. **C.** Example during asymptotic performance when the rat paused to groom before making the terminating action in the sequence. **D.** Representative example of the continuous (unaveraged, nonconcatenated) dopamine concentration v. time trace for individual sequence performance from each phase of training from a single rat that displayed the negative dopamine concentration v. time linear regression profile at asymptotic performance. Insets: background-subtracted CV from the dopamine peak for each trace; *x*-axis scale bar indicates 0.5 V and the *y*-axis scale bar indicates 0.5 nA. **E.** Dopamine concentration change during action sequence performance (at asymptotic performance- training session 5) averaged across all trials and

across subjects (shading reflects between-subject SEM) divided for rats that showed a positive v. those that showed a significant negative linear regression coefficient in the average dopamine concentration v. time trace at asymptotic performance. Although both groups of rats show elevated dopamine during sequence performance, the profile of such signaling differed. The majority of rats showed a gradual elevation in dopamine beginning prior to the sequence initiation continuing during sequence execution and reward collection/consumption. In the minority of rats more discrete elevations in dopamine concentration were apparent. Electrode placement did not differ between groups in any way observable with a cresyl violet stain. There was also no significant difference between groups in the average learning rate (t_9=1.65, p=0.13) of rats showing the positive v. negative 'ramp' characteristic at asymptotic performance, average time to complete each sequence at asymptotic performance (t_9=1.09, p=0.30), or the average amplitude of the dopamine response to unexpected reward delivery (t_9=1.60, p=0.14). The differences in these profiles may result from the heterogeneity of phasic dopamine signaling found in the NAc.

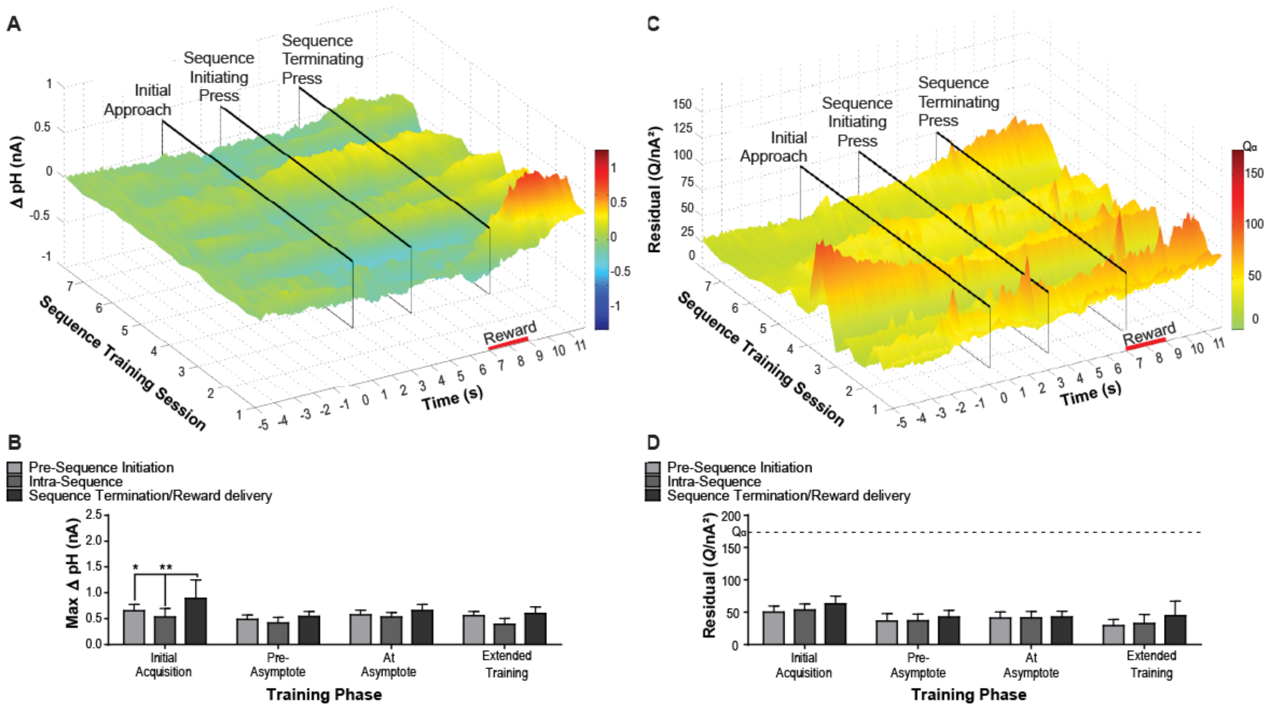

Supplementary Figure 2. Related to Figure 2. Additional output of principal component regression chemometric analysis. A&B: Analysis of the current component in the voltammetric data identified as pH by the principal component regression chemometric analysis (see Methods). **A.** Concatenated pH concentration v. time traces (see Methods) were averaged across bins of 10 sequence completions (3 bins/training session) for each rat and then averaged across rats. Time surrounding sequence performance is shown on the *x*-axis with sequence training session on the *y*-axis, pH current contribution isolated from the principal component regression analysis (see Methods) is plotted on the *z*-axis and coded in false color. **B.** Maximal pH concentration change for each task element (preceding initiating press, after the initial press but prior to the terminating press, or following sequence termination when the reward was delivered and consumed). ANOVA on these data shows no overall effect of training session ($F_{3,30}$=0.77, *p*=0.52), a significant main effect of time epoch ($F_{2,20}$=1.95, *p*=0.05) with no interaction between these factors ($F_{6,60}$=0.69, *p*=0.66). During initial acquisition there was a significant positive pH shift during the post-sequence termination epoch, but otherwise pH did not significantly fluctuate during action-sequence performance and did not show a prolonged elevation profile akin to dopamine concentration changes. **p*<0.05, ***p*<0.01. **C&D:** Analysis of residual extraneous variance in unknown measurement (*Q*) from the principal component regression. **C.** Residual *Q* values from the principal components regression (see Methods) were concatenated as described for pH above and dopamine in the main text Figure 2. Residual is

plotted on the *z*-axis and coded in false color with the 95% confidence interval tolerance ($Q\alpha$) threshold from these data set at the maximum value. **D.** Average Q values for each task element. Dashed line represents tolerance threshold ($Q\alpha$). As is clear from this figure, there is no prolonged elevation in this residual value, and the residual remains well below the tolerance for the entire sequence execution measurement interval.

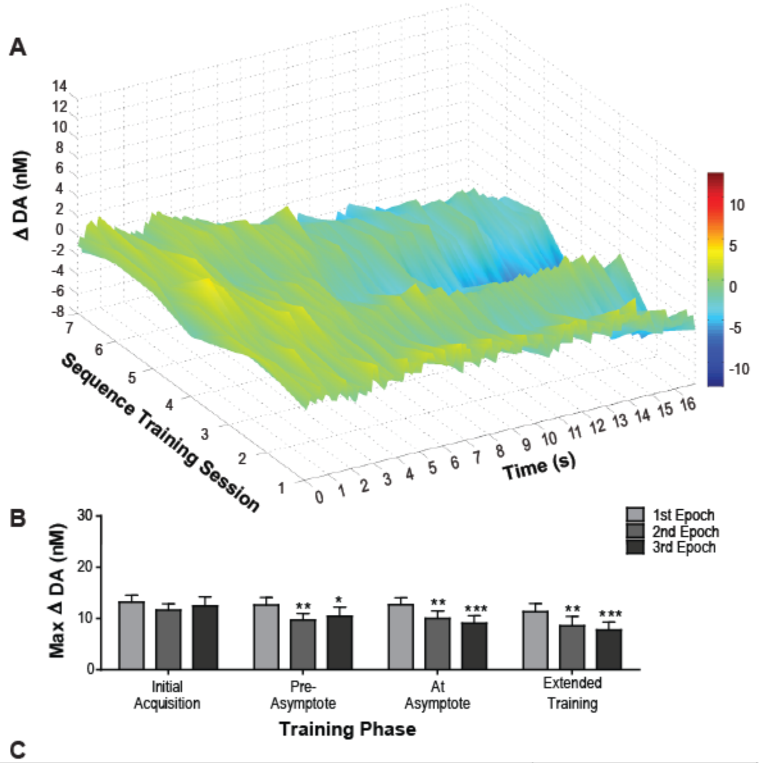

C

Training Phase	Positive Profile							Negative Profile			
Initial Acquisition	0.72	-0.32	-0.14	-0.59	0.45	-0.53	0.26	-0.65	0.68	-0.30	-0.69
Pre-Asymptote	-0.09	0.41	0.35	-0.43	-0.87	-0.54	0.23	-0.46	-0.28	-0.85	-0.84
At Asympotote	-0.82	0.31	-0.80	0.39	-0.49	-0.79	-0.87	-0.82	-0.78	-0.72	-0.81
Extended Train	-0.53	-0.66	-0.41	-0.03	-0.08	-0.84	-0.61	-0.74	-0.72	-0.84	-0.93

Supplementary Figure 3. Related to Figure 2. Dopamine concentration change during the baseline period in the absence of lever pressing activity prior to the start of the session. **A.** Dopamine concentration v. time traces (see Methods) were averaged across 5, 17-s periods (selected to match average sequence time used for Figure 2C) for each rat and then averaged across rats. Time is shown on the *x*-axis with sequence training session on the *y*-axis, dopamine concentration (nM) change is plotted on the *z*-axis and coded in false color. **B.** Maximal dopamine concentration change for each epoch of the 17-s baseline period taken to match the time window of behavioral events shown in main Figure 2C. ANOVA on these data shows no overall effect of training session ($F_{3,30}$=1.95, p=0.14), a significant main effect of time epoch ($F_{2,20}$=1.95, p<0.0001) with no interaction between these factors ($F_{6,60}$=1.19, p=0.33). Post hoc comparisons clarify that in all but the first training session peak dopamine concentration tended to show a small drift down during the baseline periods. * marks significance relative to first time epoch. *p<0.05, **p<0.01, ***p<0.001. **C.** Linear regression coefficient on the averaged dopamine concentration v. time trace during the baseline period at each phase of training for each subject. Green: positive coefficient; Red: negative coefficient.

Bolding reflects significant to at least $p<0.01$. Averaged dopamine traces were identified as having a ramping characteristic if they exhibited a significantly positive or negative linear regression coefficient (Pearson's R significant to at least $p < 0.01$). Subjects were divided by whether they showed a positive or negative ramping profile in the dopamine concentration v. time trace *during action sequence performance* at asymptotic performance (as described in Supplemental Table 1). At all phases of training a negative rather than positive ramping characteristic (Pearson's r, significant to at least $p < 0.01$) was the majority profile detected during the baseline period in the absence of lever pressing activity. These data show evidence of slight negative drift in the absence of lever-pressing activity, and support that the prolonged dopamine concentration elevations during action sequence performance were not due to chance or positive baseline drift at the electrode.

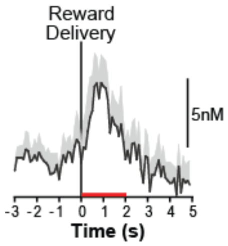

Supplementary Figure 4. Related to Figure 2. Non-contingent reward-induced phasic dopamine release. Non-contingent delivery of the orange-flavored 12.5% sucrose reward (indicated by the red bar) elicited an increase in NAc dopamine concentration. Graph represents the dopamine concentration v. time trace surrounding reward delivery averaged across trials for each rat then averaged across rats. Shading represents +1 between-subject SEM. The average maximal dopamine concentration change within the 5 s following reward delivery (which occurred at slightly variable times for each rat) was 18.67nM (SEM=1.54).

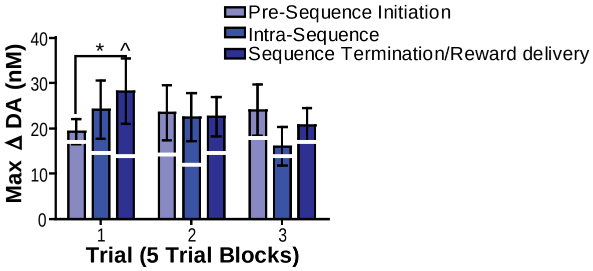

Supplementary Figure 5. Related to Figure 7. Dopamine signals across epochs of the reward value expectation violation. Rats were given a test in which the value of the reward was unexpectedly higher (orange-flavored 20% v. 12.5% sucrose solution). These data present the maximal dopamine concentration change for each task element divided into 3 blocks of 5 sequence-completion trials each during the course of the value violation test. White lines over each data bar represent the mean maximal dopamine concentration change to each task element for similar epochs during the preceding control training session. During the value violation test there was a marginally insignificant Trial block x Sequence element interaction ($F_{4,20}$=2.36, p=0.08) with the amplitude of dopamine being larger during delivery/consumption of the unexpectedly larger reward (p<0.05) than prior to sequence initiation during the first trial block. During this first trial block dopamine concentration was only significantly elevated relative to the pre-test control session during delivery/consumption of the unexpectedly larger reward (p=0.02), and not prior to (p=0.93) or within (p=0.13) sequence performance. These data suggest that early in the expectation violation test dopamine was only elevated relative to the pre-test control session during consumption/delivery of the unexpectedly higher value reward and that with subsequent exposures dopamine became elevated to more distal task elements. Error bars indicate +1 SEM. *p<0.05. ^p<0.05 relative to control session (white bar).

Training Phase	Positive Profile							Negative Profile			
**Initial Acquisition**	**-0.25**	0.10	0.10	**0.57**	**0.84**	**0.93**	**0.82**	0.06	**0.80**	**0.23**	**0.74**
**Pre-Asymptote**	**0.79**	**0.71**	-0.03	**0.77**	**0.45**	**-0.55**	**0.87**	-0.03	**0.40**	**-0.32**	**-0.48**
**At Asymptote**	**0.59**	**0.85**	**0.47**	**0.73**	**0.44**	**0.47**	**0.65**	**-0.47**	-0.17	**-0.41**	**-0.60**
**Extended Train**	**0.67**	**0.30**	**-0.68**	**0.87**	**0.66**	**0.44**	-0.10	**-0.46**	-0.14	-0.04	**-0.35**

Supplementary Table 1. Related to Figure 2. Identification of the ramping characteristic in prolonged dopamine concentration changes during action sequence execution. Linear regression coefficient on the trial-averaged dopamine concentration v. time trace at each phase of training for each subject. Green: positive coefficient; Red: negative coefficient. Bolding reflects significant to at least $p<0.01$. Trial-averaged dopamine traces were identified as having a ramping characteristic if they exhibited a significantly positive or negative linear regression coefficient (Pearson's R significant to at least $p < 0.01$) over the entire action sequence period. Subjects are divided by whether they showed a positive or negative ramping profile at asymptotic performance. At each phase of training, if a subject's data showed significant positive ramping characteristic in the trial-averaged trace, then at least 50% of the individual trials for that session also showed the same profile. Average (across subjects) proportion of individual trial dopamine concentration v. time traces for subjects that displayed the positive ramping profile in the trial-averaged trace: Training Session 1: 73.68% (SEM=2.34), 2: 66.00% (2.38), 3: 72.47 (1.49), 4: 62.22% (3.57), 5: 67.78% (9.88), 6: 70.57% (4.11), 7: 53.33% (1.11).

	Pre-Test Consumption	Preference Ratio v. Test DA Correlation			Average Sequence Time (s)	
	(g)	Sequence Initiation	Intra-Sequence	Reward Delivery	Retrain (Control)	Violation
**12.5% Orange Sucrose**	6.74	n/a	n/a	n/a	n/a	n/a
(Control)	(1.49)	n/a	n/a	n/a	n/a	n/a
**20% Orange Sucrose**	16.66**	-0.30	-0.30	-0.36	4.22	3.68
Value Increase)	(3.05)	*0.62*	*0.71*	*0.55*	(0.64)	(0.93)
**Grape**	3.65	0.25	0.25	0.05	2.39	3.20
(Alternate Flavor)	(0.78)	*0.75*	*0.49*	*0.95*	(0.79)	(0.85)
**Polycose**	11.18	-0.69	-0.69	0.41	3.50	3.09
Alternate Caloric Liquid	(4.44)	*0.31*	*0.26*	*0.59*	(0.51)	(0.42)
**Pellet**	7.19	-0.40	-0.40	-0.79	2.59	3.95
(Alternate Food)	(1.09)	*0.6*	*0.27*	*0.21*	(0.26)	(0.56)
**Water**	3.53	0.29	0.29	-0.50	2.81	4.57*
(Non-Food Reward)	(1.39)	*0.71*	*0.6*	*0.5*	(0.36)	(1.08)

Supplementary Table 2. Related to Figures 6-8. Pre-test consumption and behavioral effects of alternate rewards. Prior to all training and test, rats were exposed to the to-be trained reward (orange-flavored 12.5% sucrose) and the alternate rewards to be used for the expectation violation tests to ensure these items were not novel on test and to evaluate their relative consumption. Values in the left column represent consumption in grams (g) during these 15-min consumption tests with SEM below in parentheses. There was an overall main effect of reward type ($F_{5,23}$=4.24, p=0.007). Rats consumed more of the orange-flavored 20% sucrose (higher value reward; **p<0.01), but did not consume significantly more or less of the rewards that were different in identity (p>0.05 in all cases), indicating, along with the data in Figure 6 that these rewards were of relatively equal value to the orange-flavored 12.5% sucrose that served as the training outcome. From these data we derived a relative preference ratio for each alternate reward type [amount of alternate reward consumed g/(amount of alternate reward consumed + amount of orange-flavored 12.5% sucrose consumed g)] and correlated this against dopamine (DA) signaling during the expectation violation test. We focused on the peak dopamine concentration change prior to sequence initiation, after sequence initiation, but before the terminating lever press (intra-sequence) or upon delivery of the unexpectedly different reward. These results are plotted in the middle three columns with the Pearson r on top and the *p* value in italics on bottom. In no case was there a significant correlation between subjective

preference for the alternate identity reward and action sequence- or reward-related dopamine concentration changes. These data show that, although there was some variability in the preference ratios for the alternate rewards (especially polycose and food pellets) relative to the orange-flavored 12.5% sucrose reward (see Figure 6B), this did not influence dopamine signaling, or the lack thereof, during the expectation violation test. The 2 right-most columns show the average sequence completion time (time from initiating lever press to reward collection) during each expectation violation test and the immediately preceding re-training control session (during which the previously-trained orange-flavored 12.5% sucrose reward was earned). When controlling for multiple comparisons, sequence performance was only altered during the test in which rats earned water (18 hr water deprived) relative to the preceding retraining session (*$p<0.05$). No other manipulation significantly impacted performance ($p>0.05$ in all cases). Importantly, that changing the identity of the reward to a non-food substance had a detectable effect on sequence performance confirmed that the lack of an effect of the identity manipulations on dopamine concentration was not due to ineffective behavioral manipulations.
